# Motivation, Self-Concept and Discipline in Young Adolescents Who Practice Rhythmic Gymnastics. An Intervention

**DOI:** 10.3390/children7090135

**Published:** 2020-09-14

**Authors:** Gabriel González-Valero, Félix Zurita-Ortega, José Luis Ubago-Jiménez, Pilar Puertas-Molero

**Affiliations:** Department of Didactics of Musical, Plastic and Corporal Expression, University of Granada, Campus de Cartuja, 18071 Granada, Spain; ggvalero@ugr.es (G.G.-V.); felixzo@ugr.es (F.Z.-O.); jlubago@ugr.es (J.L.U.-J.)

**Keywords:** motivation, physical self-concept, discipline, flexibility, rhythmic gymnastics

## Abstract

This study aims to develop an intervention based on TARGET strategies in young people practicing rhythmic gymnastics, with the aim of observing whether motivation, discipline, self-concept and flexibility are improved. This research is a longitudinal study of a quasi-experimental nature. A total of 104 young adolescents between the ages of 11 and 12 years (11.66 ± 0.47) participated in the study, of which 60 belong to the control group and 44 to the experimental group. The intervention programme lasted two months (17 sessions). TARGET strategies were applied to the experimental group during training. While the experimental group continued with its routine training. To measure the psychological variables, the instrument used were the Youth Physical Self-Concept Scale (C-PSQ), Reason Scale for Discipline (RSD) and Success Perception Questionnaire (SPQ), and for flexibility, the tests were applied to the Sit and Reach and Deep trunk flexion test. The results showed that those teenagers who participated in the intervention, obtained an increased climate task, which entails an enjoyment by the practice of physical activity itself, more optimal levels of physical self-concept and discipline, subsequently, obtaining better results of flexibility. While in the control group gymnasts the ego climate and demotivation increased. TARGET strategies applied to young adolescents have positive effects, improve motivation towards physical activity, self-concept and discipline. This results in greater performance in flexibility. This will encourage young adolescents to continue to engage in physical activity in the future.

## 1. Introduction

The school has a fundamental role in the integral development of the student, whose priority is to help the student in his academic, social and personal learning [1,2]. Young adolescents spend most of their time outside this institution, and attention needs to be paid to what they do during those hours [3]. Authors such as Coulangeon [4] and Haghighat et al. [5], showed that this free time should be occupied by extracurricular activities, which are beneficial for increasing social skills, self-concept, responsibility, decreasing negative behaviours and developing healthy habits in adolescents [6,7,8,9].

In order for extracurricular activities engender adherence to physical activity and provide expected benefits to adolescents, all participants must feel empowered to carry out the activities in a satisfactory way in order to improve responsibilities [10,11]. These skills are essential so that in later stages, young adolescents can maintain an organised lifestyle [12]. Among the extracurricular activities, rhythmic gymnastics is a very popular sport among girls [13]. This sport requires a high intensity of training, dedication of many hours a week, acquisition of technical aspects through repetition of skills, high discipline and mastery of several equipment [14,15].

Regarding the psychological aspects that influence this sport, authors such as Donti et al. [16], Koumpoula et al. [17] and Oon Ai-Tan et al. [18], argue that this extracurricular activity can influence orientation, self-confidence, as well as self-concept and self-esteem. These factors can lead to reduced athletic performance, dissociative attitudes and dropouts [19,20].

The physical self-concept understood as the image that we create about ourselves, formed by the ideas that we believe define us physically, and is one of the most relevant aspects within this sport modality [21,22]. Since body image is an essential element to determine long-lasting health behaviour patterns in adolescents [23]. If this psychological aspect is not worked on properly, it will generate negative results, which can lead to eating disorders [24]. Since adolescence is a critical period, characterised by the development of feelings of body dissatisfaction, mostly in the female gender, even when the weight is below standardised levels [25].

Adolescents who have ever participated in sports associated with body expression, such as rhythmic gymnastics, ballet or swimming, show greater concern for their body weight and worse physical self-concept than those who have participated in other extracurricular activities or are physically inactive [26,27]. This has a negative impact on motivation, increasing the feeling of failure and the distance from the sports context [28].

Another aspect that influences this sport modality is motivation, since it is the source that encourages subjects to continue participating in an activity [29]. Motivational climate is understood as the set of cognitive, biological, social and emotional variables that determine the choice, intensity, resilience and performance that adolescents will spend in the development of an activity [17,30,31]. Young adolescents who participate in this sport often have ego-oriented motivation, which can be caused by emotional stress [32]. Jaenes et al. [33] also emphasise that there is a positive relationship between climate motivation, positive experience and sports performance.

In this sense, the role of the coach is essential to create a comfortable environment, where a task-oriented motivational climate is promoted, where mistakes are seen as part of the training, and fun is achieved by the practice of the activity itself and not by the results derived from it [34,35]. Another determining factor in the sports field is the predisposition to be disciplined, understood as the coordination of conducts to be instructed in order to develop sports techniques and skills, a state closely linked to the achievement of the objective [36].

Authors such as Martínez et al. [37], González-Cutre et al. [38] and Moreno et al. [39] state that discipline is linked to task-oriented motivation, to the performance of sports practice for pleasure, to the taste for learning in oneself, as well as to cooperation as a means of encouraging the team. However, ego orientation is associated with undisciplined behaviour, disobedience and demotivation.

In view of the problems faced by young adolescents participating in rhythmic gymnastics and the need to act on them, a programme was developed based on task (designing activities), authority (decision-making), recognition (rewards), grouping (working groups), evaluation (assessment criteria) and time (pace of learning and teaching), hence the acronym TARGET [40,41]. Hypotheses are proposed in relation to the intervention programme developed through TARGET strategies.

**Hypothesis** **1.**  *Pre-test data will show ego-oriented motivation, low physical self-concept, and low feelings of discipline.*

**Hypothesis** **2.**  *After the intervention with the programme, the improvement of the psychosocial aspects will have an effect on the improvement of flexibility.*

**Hypothesis** **3.**  *The implementation of TARGET strategies will have a positive impact on psychosocial variables, an increase in the motivational orientation towards the task climate, an improvement in body perception and, with it, an improvement in sports discipline.*

Therefore, in view of this problem in relation to psychosocial factors, the aim of this study is to determine whether the application of TARGET strategies promotes an increase in psychosocial variables and, thus, the physical condition of young adolescents who practice rhythmic gymnastics as an extracurricular activity.

## 2. Materials and Methods

### 2.1. Sample and Study Design

This is a quasi-experimental longitudinal study with control and experimental group. A total of 126 subjects were selected who participated in extracurricular activities of rhythmic gymnastics in different schools in Granada (Spain). The inclusion criteria considered were those participants who performed rhythmic gymnastics as an extracurricular activity, excluding those who belonged to federated clubs and those who did not complete all the intervention process. Finally, 104 girls between 11 and 12 years old (11.66 ± 0.47) participated in this research, as 8 competed semi-professionally and 14 did not complete all the sessions of the intervention programme. A simple random sampling was done to assign participants to the different groups, where 44 girls (42.3%) were in the experimental group and 60 (57.7%) in the control group.

### 2.2. Instruments

Validated instruments were used to assess young people’s physical self-concept, motivational orientation and reasons for discipline in rhythmic gymnastics. Additionally, a deep trunk flexion test and sit and reach tests were used to evaluate the flexibility of the participants.

“Sociodemographic Questionnaire”. This instrument was used to measure age, sex and type of physical-sports activity (federated or not federated), according to the categorisation proposed by González-Valero [42].

“Youth Physical Self-concept Scale (C-PSQ)”. This instrument comes from the original version of Fox et al. [43], adapted into Spanish by Moreno et al. [44]. This 28-item scale has an 11-point Likert scale ranging from “Totally Disagree” (0) to “Totally Agree” (10). This instrument has a multidimensional perspective that is grouped into four dimensions: perceived competence (items 1, 2, 10, 11, 14, 15, 16, 20, 21, 25) (Example: “I always keep in excellent physical shape”), physical attractiveness (items 3, 7, 12, 17, 22, 24, 26) (Example: “I have difficulty maintaining a beautiful body”), physical strength (items 4, 8, 13, 18, 23, 27) (Example: “Compared to most people of the same sex, I think I lack physical strength”) and self-confidence (items 5, 9, 19, 28) (Example: “I am very proud of who I am and what I can do physically”). The Cronbach consistency coefficient for the subscales was acceptable: perceived competence (α = 0.87), physical attractiveness (α = 0.76), physical strength (α = 0.67) and self-confidence (α = 0.77).

“Reason Scale for Discipline (RSD)”. This instrument was created by Papaioannou [45] and was validated into Spanish by Moreno et al. [46]. This scale has 26 items, in which the answers were indicated on a Likert scale of 11 points set at a strong disagreement (0) and a strong agreement (100), in order to facilitate the students’ response. Through this questionnaire the reasons for responsibility can be measured (items 1, 5, 10, 12, 14, 16, 17, 18, 21) (Example: “It is important for me to pay attention in class”), intrinsic reasons (items 7, 8, 19, 26) (Example: “I enjoy classes”), reasons for intrinsic regulation (items 4, 11, 25) (Example: “I will feel bad if I am not disciplined”), reasons for concern (items 6, 13, 24) (Example: “I like to help my colleagues) and reasons for demotivation (items 3, 9, 23) (Example: “I don’t understand why I should be disciplined”). The factor structure of the questionnaire shows a reliability of α = 0.86 for responsibility reasons, α = 0.76 for intrinsic reasons, α = 0.71 for intrinsic regulation, α = 0.58 for reasons of concern and α = 0.66 for demotivation.

“Success Perception Questionnaire (SPQ)”. This questionnaire was elaborated by Roberts et al. [47] and validated in Spanish by Cervelló et al. [48]. Its objective is to evaluate the dispositional orientation of the achievement goals in the sports context. This instrument has 12 items measuring the dispositional goal orientation to task (items 3, 4, 7, 8, 10, 11) (Example: “When practicing sport I feel successful when I work hard”) and dispositional goal orientation to the ego (items 1, 2, 5, 6, 9, 12) (Example: “When I practice sports I feel that I am successful when I am the best”). This questionnaire uses a Likert type scale that has a response range of 0 (Totally disagreed) and 100 (Totally agreed). The internal consistency of the questionnaire was α = 0.82 for task orientation and α = 0.91 for the ego.

“Sit and reach”. This test measures the flexibility of the lumbar area, knee flexor muscles and hip extensors [49,50]. During the execution, the subject is seated on the floor with legs together and extended. Additionally, the subjects will put their feet glued to the measurement box, with the arm extended and one hand resting on the other and facing forward. At the researcher’s signal, the subject will bend the trunk forward, pushing the cursor with both hands until the greatest distance is achieved.

“Deep trunk flexion test”. It is a test that evaluates the overall flexibility of the trunk, lower and upper limbs [51]. The barefoot subject is located within the measuring zone, positioned above the meter. The heels must be glued to the separation zone that indicates the zero point of the measurement. At the researcher’s signal, the subject flexes the legs and introduces the body between them. The extended arms are directed backwards to push the meter strip as quickly as possible, not allowing the fingers to separate from the ground and maintaining balance throughout the test.

Both physical fitness tests allow flexibility to be assessed as one of the most important elements in sports performance [49]. In addition, the subjects had two attempts to perform the tests, noting the best record of the two performed.

### 2.3. Procedure

The collaboration of the educational centres was requested through a sports company of extracurricular activities in Granada (Spain), exposing the objective of the study and requesting the cooperation of the company through an informative letter elaborated from the Area of Corporal Expression of the University of Granada. In addition, an informed consent form and the approved Ethics Committee were included. After this consent, authorisation was given to the legal tutors of the young athletes, where participation was requested on a voluntary basis and the anonymity of the study subjects’ data was ensured.

The data collection was carried out during the timetable of the extracurricular activities. The “PRE” phase took place during March, the intervention programme was carried out during the months of March and April and finally the “POST” data were collected at the beginning of May. The researchers informed the schoolchildren and directed the programme, being present throughout the process to ensure its correct application. The Helsinki Research Ethics Agreement was also respected, and permission was obtained from the Research Ethics Committee of the University of Granada (462/CEIH/2017).

The intervention programme lasted two months and consisted of 17 sessions. There were two sessions of two hours each, one per week. The programme was distributed in three phases as shown in Table 1.

The development of the programme consisted of exercises aimed to improve psychosocial considerations, including discipline, motivation and physical self-concept. To this end, the implementation was divided into three phases, which are detailed below:

In the control group, researchers were only present on the first and last day to apply the questionnaires and physical tests, where participants continued their usual training based on direct instruction for two months (warm-up based on muscle elongation and joint mobility, series of jumps, turns, and board assembly).

In the experimental group, researchers were present throughout the process. Together with the trainer, sessions were developed in which new variables were included in order to work psychosocial aspects of young athletes, as shown below.

-**Phase 1:** formed by a single session in which questionnaires and physical tests were applied to all participants in order to know the level of physical self-concept, discipline, motivation and flexibility.

-**Phase 2 (15 sessions):** In order to work on discipline, physical self-concept and subject motivation, TARGET strategies were used as a reference [23,41].

The design of tasks was modified, including tasks focused on the focus of interest of the participants. Similarly, an individualised diary was used for each subject, so that everyone would receive feedback from their trainer on a weekly basis and the objectives to be achieved would be established in the following sessions. Additionally, the diary had a section that promoted awareness of the body and the ideal weight for each depending on age, height and physical training.

In terms of authority management, transformational leadership behaviours were developed. The purpose was to stimulate the students intellectually, to encourage individual and collective communication, to inspire and motivate the participants to make an effort and carry out the proposed activities correctly.

Based on the teamwork, cooperative activities were designed to foster empathy, improve social relations and increase motivation oriented to the climate task. In addition, the subjects were encouraged to participate in the decision making and establishment of the next objectives to be achieved. Mistakes were worked on as part of the training process and not as a punishment.

For the evaluation, the subjects were made participants in it, which was focused on mastery and discipline. At the end of each week each participant was provided with a rubric in order to self-assess the level of achievement of the proposed training objectives. The coach also remarked in the individualised diary on the differences and congruencies between the opinions of young teenagers.

In terms of time management, the coach’s warm-up guidelines were followed. An attempt was made to offer individualised time for feedback and to determine technical aspects of each task.

**-Phase 5:** consisting of a single session in which questionnaires and physical tests were re-applied to all participants (control and experimental group) in order to check the changes produced by the intervention.

### 2.4. Statistical Analysis

The participation rate was 82.54% with a total of 22 invalidated questionnaires, because they did not meet the established criteria or did not develop the tests correctly. The statistical study of the parameters was executed with the statistical software IBM SPSS^®^ 24.0 (IBM Corp, Armonk, NY, USA). First, the Kolmogorov-Smirnov test was performed to establish the normality of the data. The T-student test were used to find out the differences between groups. In the mean comparison study, data were treated using a range of one to five points.

The magnitude of differences (effect sizes) was obtained while using the standardised measure Cohen’s d (*d*) interpreted as: null (0–0.19), low (0.20–0.49), moderate (0.50–0.79), or high (≥0.80) [52]. The negative sign in the effect size index means that there is a decrease, while the positive sign refers to an improvement in values. Confidence interval to 95% for each effect sizes was calculated.

## 3. Results

Table 2 shows the comparison of the sit and reach test and the deep flexion of the trunk before and after the execution of the intervention programme for the control and experimental group. In the control group, a light increase was observed in the score obtained in the sit and reach test (19.05 ± 3.09 vs. 19.25 ± 2.95) and the deep bending of the trunk (21.15 ± 2.72 vs. 21.50 ± 2.45), showing statistically significant differences for both cases (*p* = 0.027 and *p* = 0.010, respectively). According to Cohen [52], the control group had no effect on sit and reach (d = 0.06) and deep trunk flexion (d = 0.13). In the case of the experimental group, a significant increase in scores was noted in both cases (*p* = 0.000). For sit and reach (18.59 ± 3.22 vs. 20.27 ± 3.21) and deep trunk flexion (28.72 ± 3.31 vs. 30.77 ± 3.28), the programme also produced a moderate effect in both tests (d = 0.52 and d = 0.62, respectively).

Table 3 shows the comparison of the physical self-concept and its dimensions Pre-Post intervention for both groups. In the case of the control group, a decrease in the scores obtained in the General Physical Self-concept (GPSC) was observed (2.81 ± 0.22 vs. 2.73 ± 0.23), Perceived Competence (PC; 2.84 ± 0.26 vs. 2.77 ± 0.25), Physical Attraction (PA; 2.66 ± 0.66 vs. 2.57 ± 0.52), Physical Strength (PS; 2.50 ± 0.45 vs. 2.08 ± 0.42) and Self-confidence (SC; 3.70 ± 0.46 vs. 3.55 ± 0.48), all results being statistically significant (*p* < 0.05). Likewise, a null effect is highlighted for PA (d = −0.15), low effects for GPSC (d = −0.35), PC (d = −0.27) and SC (d = −0.32), being of high effects in PS (d = −0.96).

For the experimental group, an increase in the GPSC (2.81 ± 0.20 vs. 2.96 ± 0.13), PC (2.95 ± 0.30 vs. 3.38 ± 0.28) and SC (3.21 ± 0.34 vs. 3.35 ± 0.32) with a significance of *p* < 0.05, highlighting the low positive effect on SC (d = 0.40) and high for GPSC (d = 0.88) and PC (d = 1.48). However, with a level of significance of *p* = 0.000, there is a decrease in the PA (2.69 ± 0.40 vs. 2.34 ± 0.36), where the intervention has had an average negative high effect (d = −0.92).

Table 4 shows the comparison between the motivational orientation towards the task and the ego before and after the intervention programme for the control and experimental group. The control group shows statistically significant differences (*p* = 0.014) for the reduction of the task oriented motivational climate (4.00 ± 0.28 vs. 3.92 ± 0.30), offering, in turn, a low negative effect (d = −0.27). In the experimental group, an improvement was observed in the score obtained for the task climate (3.90 ± 0.15 vs. 4.28 ± 0.32), showing statistically significant differences (*p* = 0.000) and a high positive effect (d = 1.52).

Finally, Table 5 shows the comparison of the reasons for the Pre-Post treatment discipline for the established groups. In the case of control group, statistically significant data were observed (*p* < 0.05), where the general discipline reasons (GDR) (3.36 ± 0.25 vs. 3.57 ± 0.75) and reasons for intrinsic regulation (RIR) (3.11 ± 0.49 vs. 3.26 ± 0.48) increase with a low positive effect (d = 0.37 and d = 0.31, respectively). With a level of significance of *p* = 0.044, there is a decrease in reasons for responsibility (RR) (3.97 ± 0.67 vs. 3.47 ± 0.34), with a high negative effect (d = −0.94).

In the experimental group, for the reasons of discipline in general and all its dimensions, statistically significant results were obtained at the level of *p* = 0.000. After the intervention there was an increase of the values in the GDR (3.38 ± 0.14 vs. 4.06 ± 0.27), RR (3.43 ± 0.25 vs. 4.32 ± 0.42), IR (3.90 ± 0.23 vs. 4.59 ± 0.35), RIR (2.98 ± 0.48 vs. 3.78 ± 0.36) and RC (3.01 ± 0.37 vs. 4.27 ± 0.41), while reasons for demotivation (RD) (2.83 ± 0.58 vs. 2.30 ± 0.30) decreased. It should be noted that the intervention programme occasioned a high positive effect on reasons for concern (RC) (d = 3.22), GDR (d = 3.16), RR (d = 2.57), IR (d = 2.33) and RIR (d = 1.88). However, a high negative effect was obtained on the RD (d = −1.14).

## 4. Discussion

This study is similar in nature to others conducted in the population of young adolescents participating in extracurricular activities [53,54], where TARGET strategies are one of the most commonly used methods today to foster motivation in subjects. For this reason, the aim of this study has been to test the effects of an intervention programme on adolescent girls who practice rhythmic gymnastics, on physical self-concept, motivation, discipline and physical condition measured through flexibility [14,17,32,55].

The subjects who have not been immersed in the programme have increased the levels of flexibility of the areas that involve sit and reach and deep trunk flexion tests, although, according to Cohen [52], the intervention in this group that has continued with its routine training has a null effect. In this sense, similar data are detected in the study developed by Shiguemitsu-Suzuki et al. [56], in which, after applying the same flexibility tests, the control group obtained a minor improvement. This effect is due to the fact that these subjects, even though they did not participate in the intervention, have continued to train, through which it is hoped that they will improve this physical quality.

On the contrary, the intervention programme for the experimental group had a positive low-average effect [52], improving the values of flexibility. Likewise, the implementation carried out by Hsuan et al. [57], on a sample of young adults, showed that the simple fact of practicing these two tests three times in 72 h, caused an increase of this physical quality. However, the study carried out by Tisular et al. [58], in a population of young adolescents, observed that, after applying a physical activity intervention, no improvement was obtained for this physical capacity. These authors justified their results in the affirmation that the execution of physical exercises increases demotivation and the sedentary leisure acquires a major occupation of the time in such cases.

The athletes belonging to the control group decreased their perception of the physical self-concept and all its dimensions. In particular, the perceived competition, physical strength and self-confidence are undermined by the negative low-average effect [52] produced by the programme. Gatti et al. [59] and Kosmidou et al. [26], determined that feelings towards physical self-concept are negatively influenced by the drastic bodily changes that occur at these ages, pressure from the family and the trainer, competitions and excessive concern to stay thin [60,61]. This would agree with the data found in this study, since the approach of the championships intensifies the trainings and, with it, the pressure to which they are submitted, carrying consequences in the self-esteem, the physical appearance and the security in oneself in general [62].

The gymnasts who participated experimentally in the programme increased the values of perception of general physical self-concept, perceived competence and self-confidence. However, there was a decrease in the perception of physical attractiveness. In this sense, McGregor et al. [63], developed an intervention in preteens with the aim of improving self-concept through the practice of physical activity and positive feedback. With this intervention they managed to increase the feeling of competence and perceived self-esteem. Similar data were found by Wright et al. [64], which improved self-confidence and self-esteem in adolescents through the use of positive feedback and physical activity. Since the positive feedback from the coaches favours the creation of a pleasant classroom climate, which generates that the participants feel more confident of their skills and more motivated.

In terms of motivational orientations, in the control group participants the motivation for the task decreased. While this increased in the experimental group. For which the programme offered a medium positive effect [52]. Data similar to those obtained in the study by Álvarez et al. [32], which, after applying TARGET strategies, achieved an improvement in the orientation to the climate task, while slightly reducing the values of the climate ego. In the same line, Brustio et al. [65] intervened in a sample of young adolescents, where they achieved that the experimental group achieved higher levels of motivation towards the task and, with it, a greater adherence to the practice of healthy physical activity, while the control group highlighted ego orientation.

Around discipline, the control group improved general discipline and intrinsic regulation, although there was a low positive effect [52]. In addition, in these gymnasts, the reasons for being responsible decreased. As confirmed by González-Valero et al. [66] (2018) and Zaff et al. [67] (2003), regular physical activity helps young people begin to regulate themselves, although it does not in itself entail an increase in responsibility. This corroborates that general training contributes to improving the general discipline of gymnasts, although this does not lead to an improvement in commitment and motivation.

The gymnasts who participated in the intervention improved overall discipline, intrinsic reasons, responsibility, ability to regulate oneself intrinsically, and concern for discipline, where the programme had a medium–high positive effect [52]. Likewise, the values of demotivation after the intervention decreased. As Álvarez et al. [32] state, with the enjoyment of physical activity, motivation, discipline and responsibility increase, as well as the decrease in demotivation [68].

This study was not exempt from limitations. Although this is a novel study that includes a control and an experimental group, it would have been interesting to establish a comparison with elite gymnasts of similar ages. Thus, these results should not be generalised to the general population or to all physical or sports extracurricular activities. In addition, although flexibility has been shown to be one of the physical capacities most involved in gymnastics, other capacities, such as the strength and endurance of the muscles involved in the trunk and the extremities, should have been evaluated. Although the peculiarity of this study was to examine the effect of the TARGET programme on physical self-concept, discipline, motivation and flexibility, it would have been interesting to know the anthropometric characteristics of the gymnasts.

## 5. Conclusions

From this research, the main conclusions that have been drawn are that the implementation of TARGET strategies, improves the motivation towards the task in young adolescents. This results in better discipline and self-concept. It also has positive effects on improving flexibility and decreases demotivation. However, those participants who continued their regular training slightly increased flexibility, which is determined by the work done. Around motivation, it continued to be more ego-oriented, finding no improvement in either body perception or discipline. For future perspectives, it is proposed to carry out interventions comparing different age groups or establishing differences with elite gymnasts, including other psychosocial, variables involved in sport, and to consider anthropometric measures and physical capacities such as strength and resistance, which will allow conclusions to be drawn about the relationship with sporting competition and the self-concept of adolescents. In this way, one of the main practical applications of this study is that schools could apply this intervention methodology in different extracurricular activities, as it has been shown to improve adherence to physical and sports practice. In this way, the importance of managing programmes that contribute to adolescents’ adherence to healthy physical activity is emphasised.

## Figures and Tables

**Table 1:** Phases of the intervention programme.

	Phases 1	Phases 2			Phases 3
	Pre Test	Development of the Intervention			Post Test
Experimental Group	Questionnaires + Physical tests	Discipline Motivation Physical self-concept			Questionnaires + Physical tests
Control Group	Questionnaires + Physical tests	Habitual Training	Habitual Training	Habitual Training	Questionnaires + Physical tests
Number of sessions	1	5	5	5	1

**Table 2:** Pre-Post comparison of the flexibility tests for the control and experimental group.

Group	Variable	Test	M	SD	T	Sig.	95% CI	ES (d)
**Control**	SRT	Pre-test	19.05	3.09	2.265	0.027	[−0.512; 0.644]	0.06
		Post-test	19.25	2.95				
	DTFT	Pre-test	21.15	2.72	2.652	0.010	[−0.443; 0.714]	0.13
		Post-test	21.50	2.45				
**Experimental**	SRT	Pre-test	18.59	3.22	10.393	0.000	[0.008; 1.037]	0.52
		Post-test	20.27	3.21				
	DTFT	Pre-test	28.72	3.31	13.743	0.000	[0.104; 1.140]	0.62
		Post-test	30.77	3.28				

Note 1. Effect Size (ES); Confidence Interval (CI); Note 2. Sit and reach Test (SRT); Deep Trunk Flexion Test (DTFT).

**Table 3:** Pre-Post comparison of the physical self-concept for the control and experimental group.

Group	Variable	Test	M	SD	T	Sig.	95% IC	ES (d)
Control	GPSC	Pre-test	2.81	0.22	−4.009	0.000	[−0.938; −0.227]	−0.35
		Post-test	2.73	0.23				
	PC	Pre-test	2.84	0.26	−2.427	0.018	[−0.855; −0.306]	−0.27
		Post-test	2.77	0.25				
	PA	Pre-test	2.66	0.66	−2.458	0.017	[−0.730; 0.427]	−0.15
		Post-test	2.57	0.52				
	PS	Pre-test	2.50	0.45	−8.079	0.000	[−1.576; −0.354]	−0.96
		Post-test	2.08	0.42				
	SC	Pre-test	3.70	0.46	−3.150	0.003	[−0.901; 0.263]	−0.32
		Post-test	3.55	0.48				
Experimental	GPSC	Pre-test	2.81	0.20	5.767	0.000	[0.359; 1.420]	0.88
		Post-test	2.96	0.13				
	PC	Pre-test	2.95	0.30	13.789	0.000	[0.911; 2.053]	1.48
		Post-test	3.38	0.28				
	PA	Pre-test	2.69	0.40	−8.365	0.000	[−1.452; −0.388]	−0.92
		Post-test	2.34	0.36				
	PS	Pre-test	2.54	0.37	−1.480	0.146	[−0.680; 0.334]	−0.17
		Post-test	2.48	0.32				
	SC	Pre-test	3.21	0.34	2.140	0.038	[−0.088; 0.936]	0.42
		Post-test	3.35	0.32				

Note 1. Effect Size (ES); Confidence Interval (CI); Note 2. General Physical Self-concept (GPSC); Perceived Competence (PC); Physical Attraction (PA); Physical Strength (PS); Self-confidence (SC).

**Table 4:** Pre-Post comparison of motivational orientation for the control and experimental groups.

Group	Variable	Test	M	SD	T	Sig.	95% IC	ES (d)
Control	Task	Pre-test	4.00	0.28	−2.543	0.014	[−0.856; −0.305]	−0.27
		Post-test	3.92	0.30				
	Ego	Pre-test	4.54	0.19	1.362	0.178	[−0.390; 0.769]	0.19
		Post-test	4.58	0.23				
Experimental	Task	Pre-test	3.90	0.15	10.425	0.000	[0.946; 2.095]	1.52
		Post-test	4.28	0.32				
	Ego	Pre-test	4.65	0.16	0.551	0.584	[−0.389; 0.624]	0.11
		Post-test	4.90	3.00				

Note 1. Effect Size (ES); Confidence Interval (CI).

**Table 5:** Pre-Post comparison of discipline reasons for control and experimental group.

Group	Variable	Test	M	SD	T	Sig.	95% IC	ES (d)
Control	GDR	Pre-test	3.36	0.25	2.173	0.034	[−0.207; 0.959]	0.37
		Post-test	3.57	0.75				
	RR	Pre-test	3.97	0.67	1.891	0.044	[−1.550; −0.332]	−0.94
		Post-test	3.47	0.34				
	IR	Pre-test	3.85	0.39	1.657	0.103	[−0.437; 0.721]	0.14
		Post-test	3.90	0.31				
	RIR	Pre-test	3.11	0.49	3.755	0.000	[−0.272; 0.891]	0.31
		Post-test	3.26	0.48				
	RC	Pre-test	2.75	0.47	0.319	0.751	[−0.558; 0.598]	0.02
		Post-test	2.76	0.53				
	RD	Pre-test	2.45	0.64	1.230	0.224	[−0.532; 0.625]	0.04
		Post-test	2.48	0.65				
Experimental	GDR	Pre-test	3.38	0.14	16.546	0.000	[2.403; 3.921]	3.16
		Post-test	4.06	0.27				
	RR	Pre-test	3.43	0.25	16.665	0.000	[1.891; 3.260]	2.57
		Post-test	4.32	0.42				
	IR	Pre-test	3.90	0.23	13.910	0.000	[1.674; 2.986]	2.33
		Post-test	4.59	0.35				
	RIR	Pre-test	2.98	0.48	8.654	0.000	[1.277; 2.494]	1.88
		Post-test	3.78	0.36				
	RC	Pre-test	3.01	0.37	17.134	0.000	[2.459; 3.994]	3.22
		Post-test	4.27	0.41				
	RD	Pre-test	2.83	0.58	−5.877	0.000	[−1.694; −0.602]	−1.14
		Post-test	2.30	0.30				

Note 1. Effect Size (ES); Confidence Interval (CI); Note 2. General Discipline Reasons (GDR); Reasons for Responsibility (RR); Intrinsic Reasons (IR); Reasons for Intrinsic Regulation (RIR); Reasons for Concern (RC); Reasons for Demotivation (RD).
